# Distribution of Runs of Homozygosity and Their Relationship with Candidate Genes for Productivity in Kazakh Meat–Wool Sheep Breed

**DOI:** 10.3390/genes14111988

**Published:** 2023-10-25

**Authors:** Makpal Amandykova, Zhanerke Akhatayeva, Altynay Kozhakhmet, Tilek Kapassuly, Zarina Orazymbetova, Kanagat Yergali, Kadyrzhan Khamzin, Kairat Iskakov, Kairat Dossybayev

**Affiliations:** 1 Laboratory of Animal Genetics and Cytogenetics, Institute of Genetics and Physiology SC MSHE RK, Al-Farabi Ave. 93, Almaty 050060, Kazakhstan; makpal_30.01@mail.ru (M.A.); akhatayevazhanerke@163.com (Z.A.); altynaitg@gmail.com (A.K.); tilek.kapas@mail.ru (T.K.); orazymbetova.z@gmail.com (Z.O.); ergaly.qanagat@gmail.com (K.Y.); kairat11101988@mail.ru (K.I.); 2 Department of Molecular Biology and Genetics, Faculty of Biology and Biotechnology, Al-Farabi Kazakh National University, Al-Farabi Ave. 71, Almaty 050042, Kazakhstan; 3 Laboratory of Molecular Genetics, Kazakh Research Institute of Livestock and Fodder Production, Zhandosov Str. 51, Almaty 050035, Kazakhstan; kadirzhan64@mail.ru

**Keywords:** inbreeding, ROH, Kazakh meat–wool sheep, genes, fertility

## Abstract

Increasing the fertility of sheep remains one of the crucial issues of modern sheep breeding. The Kazakh meat–wool sheep is an excellent breed with high meat and wool productivity and well adapted to harsh conditions. Nowadays, runs of homozygosity (ROHs) are considered a suitable approach for studying the genetic characteristics of farm animals. The aims of the study were to analyze the distribution of ROHs, describe autozygosity, and detect genomic regions with high ROH islands. In this study, we genotyped a total of 281 Kazakh meat–wool sheep using the Illumina iScan^®^ system (EquipNet, Canton, MA, USA) via Ovine SNP50 BeadChip array. As a results, a total of 15,069 ROHs were found in the three Kazakh meat–wool sheep populations. The mean number of ROH per animal across populations varied from 40.3 (POP1) to 42.2 (POP2) in the category 1+ Mb. Furthermore, the number of ROH per animal in ROH_1–2 Mb_ were much higher than ROH_2–4 Mb_ and ROH_8–16 Mb_ in the three sheep populations. Most of individuals had small number of ROH_>16 Mb_. The highest and lowest genomic inbreeding coefficient values were observed in POP2 and POP3, respectively. The estimated F_ROH_ presented the impact that recent inbreeding has had in all sheep populations. Furthermore, a set of interesting candidate genes (*BMP2*, *BMPR2*, *BMPRIB*, *CLOCK*, *KDM2B*, *TIAM1*, *TASP1*, *MYBPC1*, *MYOM1*, and *CACNA2D1*), which are related to the productive traits, were found. Collectively, these findings will contribute to the breeding and conservation strategies of the Kazakh meat–wool sheep breed.

## 1. Introduction

Sheep breeding is a traditional branch of livestock breeding in Kazakhstan [1]. The availability of vast areas of pasture lands and the centuries-old experience of the Kazakh people in sheep breeding determines the priority of development of this sector in the country. Increasing the fertility of sheep remains one of the crucial issues of modern sheep breeding, since the level of productivity, the production of cheap lamb and wool, as well as the profitability of the industry, depend on its positive solution. The Kazakh meat–wool sheep have high meat and wool productivity, are well adapted to the extreme conditions of semi-desert and desert zones of south-eastern Kazakhstan. To increase the fertility of Kazakh meat–wool sheep, the rams of Romanov and Finn landrace sheep were used, and the obtained offspring were bred *inter se*. A herd of sheep with high fertility was created as a result of targeted selection and breeding. In 2011, an intrabreed multiparous type of the Kazakh meat–wool breed was approved [2,3].

One of the most promising methods of improving productivity livestock is the use of molecular genetic analysis, the significance of which has been proved by numerous studies [4]. There is also an active search for promising marker genes in sheep breeding. In this regard, the *growth differentiation factor 9 (GDF9*), *bone morphogenetic protein receptor IB (BMPRIB)*, *and bone morphogenetic protein 15 (BMP15)* genes that influence reproductive qualities are of great interest [5].

It is known that close inbreeding is happening during breed formation. It is a natural and inevitable phenomenon in animal populations [6]. Meanwhile, due to a high degree of inbreeding some genetic characteristics, such as selection signatures, specific haplotypes, selective sweeps, and selection pressure, are fixed in a population. A decrease in the productivity of inbred animals is called inbred depression [7]. In addition to providing a more powerful measure of inbred depression, genomic data offer additional opportunities for understanding the genetic background of inbreeding. Nowadays, runs of homozygosity (ROHs) are considered a suitable approach for studying the genetic characteristics of farm animals [8]. ROHs are continuous homozygous regions that are present in an animal’s genotype because both parents passed the same haplotypes to their offspring [9]. When two identical alleles at a locus come from a common ancestor through non-random mating (inbreeding), the genotype is said to be autozygous, or identical by descent (IBD). While two identical alleles come from different sources, the genotype is called allozygous, or “identity by state” (IBS). ROH methods allow for the more accurate identification of alleles at the IBD locus and are widely used in human and animal studies to accurately estimate the level of autozygosity [10]. ROH length can be used to study the effect of inbreeding age on inbred depression in addition to existing pedigree-based methods. Also, calculations using genotype information are more accurate than pedigree information. The length of the ROH pattern can be used to track the ontogeny of the autozygous segments: short ROH segments reflects an older inbreeding origin, as opposed to long ROH segments, reflecting a recent inbreeding origin [11]. For instance, calculating the genomic inbreeding levels from ROHs has shown an accurate estimator for cattle population [12].

Selection traits are the result of genotypic changes in populations subjected to some form of selection pressure [13]. These changes are characterized by an increase in the frequency of alleles in one or more genes or clusters of genes involved in the processes of population adaptation to specific conditions or in the improvement of productivity. Methods that allow for the identification of such traits may open up opportunities for the identification of genes involved in these processes [14]. Given the increasing availability of genomic information, particularly single-nucleotide polymorphism (SNP) data, genome-wide homozygosity can be better predicted and therefore the negative consequences of homozygosity compared to “pedigree” breeding can be better reflected [15]. Recently, several studies revealed genes in sheep within the ROH islands [16,17,18]. For instance, Li et al. (2022) detected several interesting candidate genes, including *BMPRIB* in famous high-prolific Hu sheep [18]. The *IGF1*, *TGFBR2*, etc. genes that affect muscle development have been reported in Chaka sheep based on the ROH analysis [19]. Also, ROH outcomes displayed distinctness because of increased inbreeding among the Spanish Merino sheep [20].

Therefore, the objective of this research was to detect ROH features in Kazakh meat–wool sheep population and select candidate genes within the ROH islands that are related to breed-specific traits in tested populations.

## 2. Materials and Methods

### 2.1. Sample Collection

A total number of 281 Kazakh meat–wool sheep bred in “Kuatzhan” (n = 95 female (n = 95♀ (POP1)) and “Dukeev” (n = 115 female (POP2) and 71 male (POP3)) farms were used in this work. The two named farms have been working on improving sheep fertility for a long time in the animal husbandry sector of our country. Therefore, it is of interest to breeders to study their productivity and fecundity. Animals were selected for the study on the basis of their fecundity. Ewes with twin or more twin births and lambs were selected for this study. The blood samples were collected by the veterinarian into EDTA-containing tubes to prevent blood clotting. All animal experiments were approved by the Local Ethics Committee of the RSE Institute of Genetics and Physiology SC MSHE RK (19 October 2021, Almaty, Kazakhstan). The specimens were transported to the Laboratory of Animal Genetics and Cytogenetics of the RSE Institute of Genetics and Physiology using the blood bank refrigerator and stored in a freezer (at −25 °C) until DNA extraction.

### 2.2. DNA Extraction and SNP Genotyping

Genomic DNA was extracted from the whole blood using a GeneJET Whole Blood Genomic DNA Purification Mini Kit, #K0782 (Thermo Scientific, Waltham, MA, USA). This kit utilizes silica-based membrane technology in the form of a convenient spin column. The standard procedure takes less than 20 min following cell lysis and yields purified DNA more than 30 kb in size. 200 μL of whole blood was mixed with 20 μL of Proteinase K Solution by vortexing. After adding 400 μL of Lysis Solution and vortexing, the tubes were incubated at 56 °C for 10 min using a thermomixer. Then, 200 μL of ethanol (96–100%) was added to the tubes and mixed by pipetting. After transferring the prepared mixture to the spin column, the tubes were centrifuged at 6000× *g* for 1 min. Wash Buffer I and Wash Buffer II in 500 μL were used to clean the purification column from unnecessary cellular debris. It is crucial to centrifuge tubes at high speed (≥20,000) after every step of the washing process. In the final step, 200 μL of Elution Buffer was added to the center of the column membrane to elute genomic DNA. To increase the output DNA concentration, it is necessary to reduce the amount of Elution Buffer added. The presence of DNA was checked by 1% agarose gel electrophoresis and the quality of genomic DNA was assessed by Nanodrop One (Thermo Scientific, Waltham, MA, USA) spectrophotometer. The DNA concentration was diluted to 50 ng/µL before genotyping. Genotyping was performed using the Illumina iScan system (EquipNet, Canton, MA, USA), which supports the rapid, sensitive, and accurate imaging of Illumina BeadChips for genetic analysis results, and the OvineSNP50 BeadChip microarray, consisting of more than 54,000 SNPs to deliver the densest coverage available for the ovine genome. Further, the ped and map format files were generated using the PLINK Input Report Plug-in parameter implemented in GenomeStudio v2.0 software. Here, the ped files contained the information on the family and sample IDs, paternal and maternal IDs, sex of the animals, and SNPs information, while the map files contained the chromosome ID, unique SNP identifier, genomic distance, and SNP position information.

### 2.3. Quality Control of Data

The primary processing of genotyped data was conducted in the GenomeStudio software. Further quality control of the data was carried out using Plink v1.07 software. The SNPs that unmapped contigs, located on sex chromosomes and mtDNA, were excluded. For the quality filtration of genotyped data, the following parameters were applied: missing genotypes >10%, SNPs with call rate <90%, the minor allele frequency *p* < 0.05, and Hardy–Weinberg equilibrium *p* < 0.0001.

For the identification and statistical analysis of runs of homozygosity, Plink v1.07 software and the detectRuns package were used. The following parameters and thresholds were applied for the identification of ROHs: the minimum ROH length was set to 1 Mb with 15 SNP, the window threshold to call a ROH was 0.05, one missing call was allowed per window, the sliding window size in SNPs was set to 15, the required minimum SNP density in an ROH was 1 SNP per 100 kb, and the maximum gap between two consecutive homozygous SNP was 250 kb.

### 2.4. Calculation of Inbreeding Coefficient

Runs of homozygosity is one of the common methods to evaluate genomic inbreeding coefficient in animals. The inbreeding coefficient on the basis of ROHs (F_ROH_) for each individual was evaluated as follows:

F_ROH_ =∑L_ROH_/L_aut_;

where L_ROH_ is the total length of all ROHs, and L_aut_ is the specified length of the autosomal genome covered by SNPs on the chip [21]. Here, we used ROHs above 1 Mb in different classes to calculate F_ROH_ for Kazakh meat–wool sheep. The literature review show that ROHS above 1 Mb are the most recurrently used to evaluate of genomic inbreeding coefficient and recent animal relatedness for farm animals.

### 2.5. Detection of ROH Islands

To determine of common ROH islands, which are the regions in which ROHS are most frequent in that population, the percentage of occurrence of SNPs falls inside ROHS was calculated, and it was plotted against SNP positions in all chromosomes. The top 1% of the highest occurrences SNPs were chosen as the threshold to identify the genomic regions most commonly associated with ROHS. For the visualization of high frequency of SNPs fall in the homozygous fragments, the Manhattan plot was generated.

## 3. Results

### 3.1. Runs of Homozygosity Analysis

**Figure 1:**
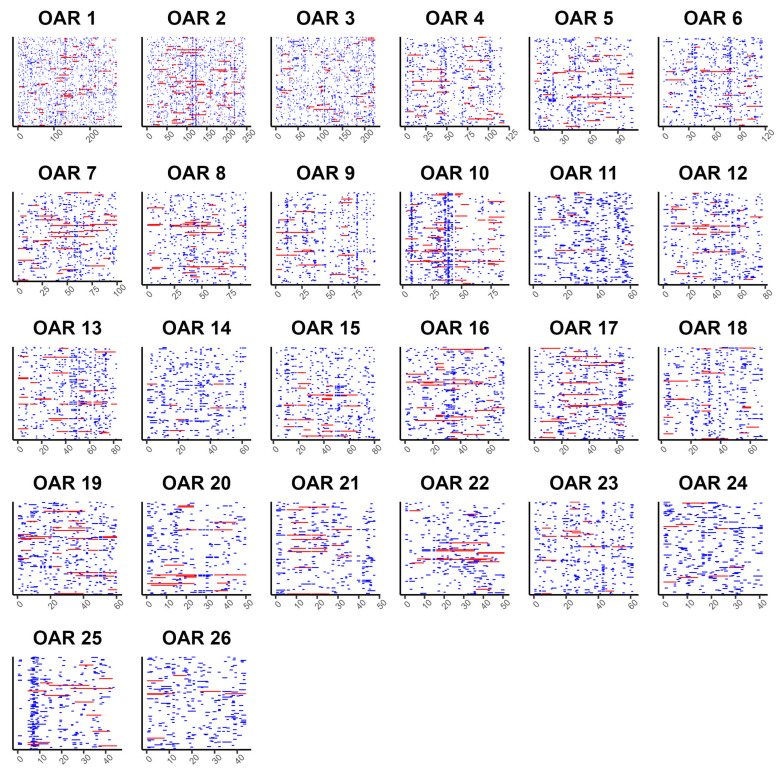
Distribution of runs of homozygosity fragments for each autosomal chromosome, with the length of OAR (*x*-axis) in Mb. Red long lines mean ROH (>5 Mb ROH), blue short lines mean (<5 Mb).

In the present study, Ovine SNP50 BeadChip array was used to characterize runs of homozygosity level in the genome of Kazakh meat–wool sheep breed. After quality control, 281 animals with 46,143 SNPs were retained for further analysis. A total of 15,069 runs of homozygosity fragments were found in the three populations of Kazakh meat–wool sheep. The distribution of ROHs across all chromosomes is displayed in Figure 1. Descriptive statistics on the number and length of ROHs in different classes are described in Table 1 for the three sheep populations. The mean number of ROHs per animal decreased with the growing length category of autozygosity in all populations. The results presented a rapid decrease in the mean number of ROHs per animal starting from 2+ Mb in the studied sheep. The average number of ROHs per individual ranged from 40.3 to 2.1 Mb at categories 1+ and 16+ in the POP1 population, while this value was varied from 42.2 to 1.6 Mb at categories 1+ and 16+ in the POP2 population. Regarding POP3, the mean number of ROHs was between 40.7 and 1.1 at classes 1+ and 16+ Mb, respectively. The estimated mean number of ROHs per animal was higher in POP2 compared to POP1 and POP3 in all categories, except 16+ Mb. The average ROH length varied from 51.4 (1+ Mb) to 17.2 (4+ Mb), values in category 8+ and 16+ Mb were higher than that of 2+ and 4+ for POP1. The highest and lowest of mean length of ROHs was observed in category 1+ and 4+ for POP2, while this value observed in classes 1+ and 4+ for POP3.

### 3.2. Genomic Inbreeding Coefficients

To evaluate of genomic inbreeding level in the three populations, the inbreeding coefficients on the basis of runs of homozygosity (F_ROH_) in different ROH length classes was estimated (Table 2). For the estimated inbreeding coefficients above 1 Mb, the highest value was observed in POP2 (0.047), with the lowest value in POP3 (0.036). According to F_ROH_ > 2 Mb, the genomic inbreeding level varied from 0.025 in POP2 to 0.014 in POP3. However, the highest value of F_ROH_ > 16 Mb was observed in POP1, and the lowest was recorded in POP3. POP3 had the lowest genomic inbreeding level in all F_ROH_ categories. In all populations, F_ROH_ was higher in >8 Mb compared to >4 Mb. The inbreeding coefficient values were stable at the last three F_ROH_ classes in POP1. F_ROH_ coefficients varied from 0.047 (>1 Mb) to 0.013 (>16 Mb) for POP2, while these values were between 0.036 (>1 Mb) and 0.008 (>16 Mb) for POP3. F_ROH_ coefficient rates rapidly declined at >2 Mb in all populations.

### 3.3. Gene Annotation

**Figure 2:**
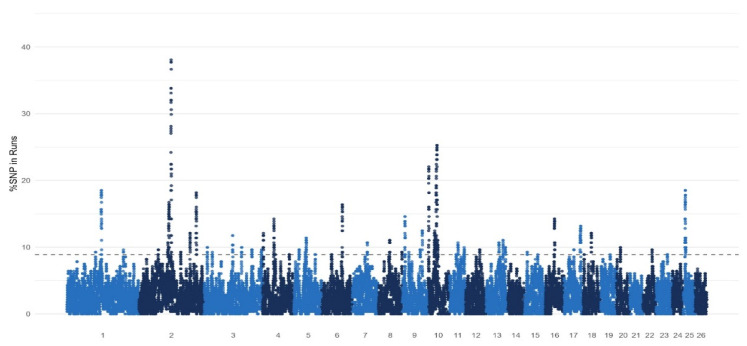
The occurrence (%) of SNPs in ROHs across individuals.

Next, to investigate effect of selection in the sheep populations, the occurrence of ROHs across genomes were explored. The Manhattan plot showed that the highest frequency of ROH occurrence was observed on 1, 2, 6, 10, and 25 autosomes. Further, the top of 1% of SNPs were selected for the studied sheep populations (Figure 2). We annotated all genes within potential ROH islands and found that the genes that were identified are related to reproductive (*BMP2*, *BMPR2*, *BMPR1B*, *CLOCK*, *KDM2B*, *TASP1*), carcass, or meat traits (*MYBPC1*, *MYOM1*, *CACNA2D1*), with the inclusion of one novel *TIAM1* gene. Table 3 displays the genes within the ROH islands.

## 4. Discussion

Controlling the level of inbreeding and maintaining adequate genetic diversity is crucial for the robustness of sheep population. The estimation of the inbreeding coefficient based on pedigree information was not accurate due to incomplete pedigrees, the small number of generations, and errors. One of the strengths of ROH analysis is the ability to reliably identify long homozygous segments even at relatively low marker densities. This makes ROHS attractive for studying and understanding the role of autozygosity and as a possible tool for managing inbreeding levels for breeding programs in livestock production [22]. Therefore, inbreeding evaluation coefficients based on genome-wide SNP markers by ROHs gives more rigorous results [23]. The number and length of ROHs can directly reflect the genetic history of a population [24].

A total of 15,069 ROHs were found in the three Kazakh meat–wool sheep populations, and the high frequency of ROHs in these populations may be due to their restricted geographical location. Furthermore, POP2 had the highest number of short ROH fragments, indicating more ancient inbreeding events. Numerous research studies have also reported the abundance of ROHs in the short-length category in other sheep breeds [25,26]. In terms of length, the average length of ROHs across population ranged from 12.5 Mb (POP3) to 54.1 Mb (POP2). The mean length of ROHs per animal (Mb) was higher in ewes compared to rams in all ROH length categories except 1+ Mb class, which is in agreement with the mean number of ROHs per animal and F_ROH_ results. The lowest values of inbreeding coefficients (0.008–0.036) in POP3 may indicate a relatively short breeding history in this population. This is in accordance with reality; after weaning, the rams usually are separated to temporary stock to buy and slaughter. Similar outcomes were found in Spanish sheep breeds [26]. Besides, ROH patterns have specificity in various chromosomes in the present study, which is consistent with previous findings [27,28].

In our study, significant homozygous regions were found in Kazakh meat–wool sheep, which may indicate that some homozygous regions are contributing to fertility complications due to inbreeding depression. The inbreeding coefficient for each of the categories (F_ROH1Mb_, F_ROH2Mb_, F_ROH4Mb_, F_ROH8Mb_, and F_ROH16Mb_) was estimated. The number of segments longer than 16 Mb is much smaller, indicating that the level of inbreeding in the studied sheep population is low, and that they are unaffected by recent inbreeding events. In general, these results are consistent with previously observed results in other sheep breeds [18,29]. On the other hand, recent inbreeding resulted in a long ROH (>16 Mb), which may indicate the ineffectiveness of recent genetic management attempts. In contrast, the abundance of short F_ROH1Mb_ indicates inbreeding that occurred in ancient times. Ancient and recent inbreeding emphasize the relevance of measures to maintain high fertility and limit inbreeding in Kazakh meat–wool sheep.

Although not all identified genes are proliferation-associated genes, some important genes have been identified in this study. It has been well documented that bone morphometric proteins (BMPs) are multifunctional growth factors belonging to the transforming growth factor β (TGF-β) superfamily, whose role in sheep reproduction has been widely studied in recent years [30]. *Bone morphogenetic protein 2 (BMP2)* plays an important role in bone formation and regeneration. BMP-2 protein is one of the most effective inducers of osteogenesis that activates the expression of osteoblast markers [31]. Of interest, polymorphisms within the *BMP2* gene were associated with tail length in Chinese Hu and Tibetan sheep [32]. *Bone morphogenetic protein receptor type 2 (BMPR2)* encodes the bone morphogenetic protein receptor type II, which belongs to the transforming growth factor receptor (TGFR) superfamily. The BMPR2 receptor regulates the number of cells in tissues by triggering and controlling their proliferation and apoptosis [33]. Importantly, it has been revealed that the expression of *BMPR2* in cumulus-oocyte complex and ovary tissues were related to the sheep antral follicle number [34]. The *BMPR1B* gene is well known to be play an essential role in sheep reproductive traits [35]. Interestingly, the *Clock* gene, which is a transcription activator, was highly expressed in the cerebellum, hypothalamus, and uterus in Small-tail Han sheep [36]. In addition, *Lysine demethylase 2B (KDM2B)* has been reported to play a role in spermatogenesis [37].

*T lymphoma invasion and metastasis protein (TIAM1)* encodes the invasion of T-cell lymphoma, and it is critical for the integrity of adherent junctions and cell–matrix interactions [38]. *Myosin regulatory light chain-2 (MYBPC1)* was associated with lamb meat quality characteristics [39]. *Voltage-dependent calcium channel subunit α-2/delta-1 (CACNA2D1)* encodes the CACNA2D1 protein, which is cleaved into the α 2- and delta-subunits of a potential-dependent calcium channel. These channels play an important role in coupling muscle excitation and contraction. The SNP mutations in this gene was significantly associated with carcass traits in cattle [40,41]. Recently, the GWAS revealed that the *Threonine aspartase 1 (TASP1)* gene was related to the fertility and precocity in Nelore cattle [42] as well as increased fiber muscle diameter in ducks [43]. Furthermore, *Myomesin-1 (MYOM1) was* found to be differentially methylated by gene ontology enrichment analysis in sheep [44].

## 5. Conclusions

In this study, we investigated the use of selection signature analysis within the Kazakh meat–wool sheep populations based on homozygosity. To the best of our knowledge, this is the first study identifying the genomic regions of a Kazakh meat–wool sheep breed. Moreover, genomic regions associated with reproductive traits as well as carcass and meat traits have been identified in tested population. The results obtained can be used in practice to improve the genomic characterization of this breed.

## Figures and Tables

**Table 1:** Descriptive statistics of ROHS for three sheep populations.

Populations		Statistics	ROH Length Category (Mb)				
			1+	2+	4+	8+	16+
POP1 n = 95	Number of ROHs per animal	Mean	40.3	6.9	3.2	2.4	2.1
		SD	6.8	3.0	2.7	1.9	1.2
		Min	26	1	0	0	0
		Max	58	15	12	8	5
	Length of ROHs per animal (Mb)	Mean	51.4	18.7	17.2	26.2	41.4
		SD	8.9	8.3	15.1	21.4	25.0
		Min	31.2	2.2	0	0	0
		Max	74.4	41.8	68.8	96.5	101.5
POP2 n = 115	Number of ROHs per animal	Mean	42.2	8.0	3.9	3.2	1.6
		SD	11.0	5.0	3.4	3.0	1.1
		Min	17	1	0	0	0
		Max	102	30	16	14	5
	Length of ROHs per animal (Mb)	Mean	54.1	21.5	21.4	35.3	31.8
		SD	14.6	14.1	19.3	33.1	22.5
		Min	22.5	2.1	0	0	0
		Max	133.5	85.2	85.6	143.7	100.2
POP3 n = 71	Number of ROHs per animal	Mean	40.7	6.4	2.3	1.8	1.1
		SD	8.5	3.5	1.7	1.1	0.4
		Min	24	1	0	0	0
		Max	83	23	7	4	2
	Length of ROHs per animal (Mb)	Mean	52.5	17.3	12.5	19.6	19.9
		SD	11.5	9.2	9.3	14.7	5.7
		Min	30.3	2.3	0	0	0
		Max	112	58.6	38.5	53.6	33.8

**Table 2:** The genomic inbreeding coefficient values within different classes.

Population	F_ROH_ > 1 Mb	F_ROH_ > 2 Mb	F_ROH_ > 4 Mb	F_ROH_ > 8 Mb	F_ROH_ > 16 Mb
Pop1	0.042	0.021	0.016	0.017	0.017
Pop2	0.047	0.025	0.019	0.020	0.013
Pop3	0.036	0.014	0.009	0.010	0.008

**Table 3:** The identified top of 1% genes within the ROH islands.

CHR	nSNP	Start, Mb	End, Mb	Candidate Genes
1	78	130.47	135.69	*TIAM1*
2	36	218.8	219.93	*BMPR2*
3	20	182.28	183.49	*MYBPC1*
4	24	42.18	43.2	*CACNA2D1*
6	21	78.04	79.31	*CLOCK*
6	33	33.61	35	*BMPR1B*
13	19	50.59	51.89	*BMP2*
13	204	0.12	8.87	*TASP1*
17	87	60.65	64.98	*KDM2B*
23	28	41.93	43.98	*MYOM1*

## Data Availability

Not applicable.
